# Bone defects following curettage do not necessarily need augmentation: A retrospective study of 146 patients

**DOI:** 10.1080/17453670902804505

**Published:** 2009-02-01

**Authors:** Martti Hirn, Uday de Silva, Sujith Sidharthan, Robert J Grimer, Adesegun Abudu, Roger M Tillman, Simon R Carter

**Affiliations:** ^1^The Oncology Unit, Royal Orthopaedic Hospital,BirminghamUK; ^2^Department of Orthopaedics, Tampere University HospitalTampereFinland

## Abstract

**Background and purpose** The natural pattern of bone healing in large bony defects following curettage alone as treatment of benign bone tumors around the knee is not well reported. We analyzed the outcome in 146 patients.

**Patients and methods** 146 patients with over 18 months of follow-up who underwent curettage without bone substitute filling or bone grafting for a benign tumor in the distal femur or upper tibia were included. The mean diameter of the defects following curettage was 5.7 (1.3–11) cm and the estimated average volume was 63 (1–240) cm^3^. The plain radiographs before and following curettage were reviewed to establish the size of the initial defect and the rate of reconstitution and infilling of the bone. The time to full weight bearing and any complications were recorded.

**Results** There was a variable rate of infilling; some defects completely reconstituted to a normal appearance while some never filled in. In 88% of the cases, no further intervention after curettage was required and the mean time to full weight bearing was 6 weeks. The risk of subsequent fracture or the late development of osteoarthritis was strongly related to the size of the cyst at diagnosis, with cysts of > 60 cm^3^ (about 5 cm in diameter) having a much higher incidence of complications.

**Interpretation** This study demonstrates the natural healing ability of bone without any adjuvant filling. It could be used as a baseline for future studies using any sort of filling with autograft, allograft, or bone substitute.

## Introduction

The management of benign bony defects has been the subject of much debate between orthopedic surgeons in recent years. There has been an increasing trend towards intraoperative filling of these lesions, and this is especially so when dealing with defects in weight-bearing areas. Large bone cavities have been reinforced with autologous bone grafts, allografts, poly(methylmethacrylate) (PMMA) bone cement, and bone substitutes (Mjöberg et al 1984, Ryan and Begeman 1984, Sethi et al. 1993, Finkemeier 2002, Malek et al. 2006). These methods may have several limitations due to the limited availability of autograft and the low risk of disease transmission associated with the use of allograft (Tomford 1995). PMMA cement may provide instant stability, but it is not the most biological method for filling of bone (Mjöberg el al. 1984). There is also concern that when used near the surface of a joint, it may cause thermal injury and damage chondrocytes (Dunne and Orr 2002). Many different bone substitutes, aimed at filling these defects (Finkemeier 2002), are flooding the market; yet, there is very little evidence for their efficacy and there have been very few comparisons with the normal degree of healing expected in bone defects (Virolainen et al. 1997, Lemperle et al. 1998, Kopylov et al. 2002). Despite the fact that there is a strong capacity to create new bone after trauma, there have been very few studies reporting on the ability of a surgically created bone defect to fill in if left empty (Medige et al. 1982, Virolainen et al. 1997, Lemperle et al. 1998). The paucity of data on the natural history of bone defects aroused our interest. In a retrospective study, we thus investigated the outcome of 146 benign bony tumors about the knee that had been treated with curettage alone without any augmentation.

## Patients and methods

We identified 146 patients with benign bone tumors about the knee who had undergone curettage without grafting or other filling of the defect. These patients had been treated at the Royal Orthopaedic Hospital, Birmingham between 1983 and 2003. The data were gathered from medical records and all the patient radiographs were examined to determine whether there had been healing of the defect or whether complications had developed.

The criteria for inclusion were a histologically confirmed solitary benign lesion around the knee, with no previous surgery treated with curettage, with no augmentation of the defect, and with at least 18 months of follow-up.

The mean age of the patients was 28 (1–71) years; 43 were under 16 years of age at the time of treatment. There were 83 males and 63 females. The average follow-up was 4 (1.5–20) years. The most common histological diagnosis was giant cell tumor in 68 patients, followed by chondroblastoma in 21 (Table 1).

**Table 1.** Histological diagnosis of the 146 tumors

	No.	%
GCT	68	47
Chondroblastoma	21	14
ABC	19	13
Enchondroma	9	6
Fibrous dysplasia	5	4
Others	24	16

Surgery involved intralesional curettage through a generous cortical window followed by burring, brushing, and pulse lavage of the lesion. After surgery every cavity was left empty without any augmentation, and drains were not inserted. Patients were asked to undertake partial weight bearing for 2–10 weeks, depending on the size of the cyst and the radiological features. All were on full weight bearing after 10 weeks.

The pre- and postoperative radiographs were examined and measurements of size and estimates of consolidation were made and documented sequentially. The presence of osteoarthritic changes or fractures was also noted.

The volume calculations were done as follows, where A = width, B = depth, and C = height.

Cylinder defect = ABC × 0.785, i.e. (π × A/2 × B/2 × C).

Spherical defect = ABC × 0.52, i.e. (4/3 × π × A/2 × B/2 × C/2).

The most appropriate of the two formulae was used, depending on the perceived shape of the defect.

Statistical significance was analyzed using the chi-squared test and Mann-Whitney U test. A value of p < 0.05 was considered significant.

The outcomes were based on radiographic consolidation of the lesions along with subjective clinical assessment and function recorded in the patient records.

## Results

129 of the cysts (88%) were within 15 mm of the surface of the joint. 104 of the cysts (71%) were located in the tibia and 42 were in the femur. A medial location of the cyst was most common in both the tibia and femur (Table 2).

**Table 2.** The anatomical location of the cysts

	Medial	Central	Lateral
Femur	22	6	14
Tibia	47	27	30

The mean maximum diameter of the cysts was 5.7 (1.3–11) cm and the mean volume of the lesions was 63 (1–240) cm^3^. The femoral cysts were larger than the tibial ones (p = 0.05) (Table 3). 64 of the cysts had a maximum diameter of 5 cm or less, while 82 had a diameter of more than 5 cm.

**Table 3.** The mean volume of the cysts (cm^3^) according to the anatomical location

	Mean	Medial	Central	Lateral
Femur	78	92	42	72
Tibia	51	49	71	54

6 patients had preoperative fractures and 14 had postoperative fractures through the cyst, all within 2 months of their operation. All 14 postoperative fractures were in patients with giant cell tumors. Only 2 fractures required internal fixation; all the others were stable fractures and were treated nonoperatively in plaster with restriction of weight bearing. There was no correlation between preoperative fracture and size of the cyst, but 4 of the 6 arose in patients with giant cell tumors and 4 arose in lateral locations—2 in the tibia and 2 in the femur. There was, however, a strong correlation between risk of postoperative fracture and both the size and volume of the cyst. The average size of the cysts that fractured postoperatively was 108 cm^3^, as compared to 58 cm^3^ for the cysts that did not fracture (p = 0.003). The risk of fracture was 5% in patients with cysts less than 60 cm^3^, as compared to 17% for those with cysts larger than 60 cm^3^ (p = 0.01). The risk was 3% when the maximum diameter of the cyst was ≤ 5 cm, but 15% when the diameter was > 5 cm (p = 0.02).

16 patients (11%) developed local recurrences during the period of follow-up, with the risk of local recurrence being 15% for giant cell tumours and 16% for aneurysmal bone cysts. 10 patients had repeated curettage, 2 required subsequent augmentation with cement, and 4 had endoprosthetic replacement.

The average time to bone healing, as judged by allowing full weight bearing, was 6 (2–10) weeks. The mean time to full weight bearing was 5.4 weeks for patients with cysts up to 5 cm in diameter and 6.7 weeks for those with cysts over 5 cm (p = 0.003). Although there was a trend for older patients to take slightly longer to progress to full weight bearing, this was not statistically significant.

None of the patients required bone grafting or a further surgical procedure unless they developed a fracture or local recurrence. Review of serial radiographs showed that while the smaller cysts filled up completely, the larger ones tended to heal initially by thickening of the cortex and then by development of septae running across the defect. This appearance remained unchanged for years.

During the course of follow-up, 16 knees showed radiographic changes consistent with osteoarthritis. 2 of these patients had preoperative pathological fractures while 2 others had postoperative fractures. 9 of the 16 patients had symptoms attributable to the osteoarthritis. 7 suffered from a persistent stiff knee with pain, and 2 suffered from chronic pain only. The rest of the patients who developed osteoarthritis were still asymptomatic at the time of the study. There was a strong correlation between incidence of degenerative changes and tumor size; these changes arose in 13 of 59 patients with a cyst greater than 60 cm^3^ but only in 3 of 87 patients with cysts smaller than 60 cm^3^ (p = 0.004). All but one of these patients who had radiographic osteoarthritis had a giant cell tumor.

The risk of structural complications (fracture or osteoarthritis) was thus strongly associated with the size of the cyst at the time of diagnosis. 24 patients of the 59 with a cyst greater than 60 cm^3^ developed either or both of the structural complications, as compared to 8 of the 87 patients with a smaller cyst (p < 0.0001).

Only 9 patients (6%)—all of whom had large cysts (> 60 cm^3^)—did not achieve good function with a satisfactory range of knee movement and no pain when walking.

## Discussion

Cure rates of 95% have been achieved using curettage as the sole treatment in benign bony lesions, although in some cases the curettage had to be repeated (Kreicbergs et al. 1985). The overall recurrence rates may vary quite considerably, depending on the histology of the disease (O'Donnell et al. 1994). In giant cell tumors treated by curettage with adjuvant filling, recurrence rates have varied between 7–50% (Blackley et al. 1999, Trieb et al. 2001, Saiz et al. 2004, Zhen et al. 2004, Prosser et al. 2005, Malek et al. 2006, Vult von Steyern et al. 2006). There have been many attempts to try to reduce this rate of local recurrence, including the use of adjuvants such as phenol, cryotherapy, and bone cement (Turcotte et al. 2002, Zhen et al. 2004, Szalay et al. 2006). Although individual centers have claimed good results using a variety of techniques, large population studies have failed to show that any one technique is better than any other and to date there have been no randomized controlled trials investigating this, even though several authors have suggested such (Capanna et al. 1985, Trieb et al. 2001, Turcotte et al. 2002, Prosser et al. 2005). The use of PMMA cement was advocated on the basis that not only would it fill the defect and supply structural support, but it might also give benefit through its thermal effect on residual tumor cells, but most large series have failed to show convincingly that local control is better with or without cement (O'Donnell et al. 1994, Turcotte et al. 2002). All authors agree, however, that adequate exposure and detailed curettage is essential to maximize local control, whether adjuvants are used or not (Blackley et al. 1999, Turcotte et al. 2002).

Curettage is also used for the treatment of other benign cysts, and the question of whether adjuvants are necessary has also been raised for both aneurysmal bone cysts and chondroblastoma (Cottalorda and Bourelle 2006, van der Geest et al. 2007).

Our policy of curettage alone without adjuvants appears justified as our rates of local control are well within those reported from other centers. We, too, believe that a randomized controlled trial should be done to try to resolve this question.

Equally contentious is the decision as to what to do with the defect that is created following curettage. There is a natural tendency to wish to fill it with something, but again there is no evidence to show what is best—and there is also no evidence to suggest that filling the defect improves the chances of local control. Our policy of leaving these cavities empty was based initially on the observation that small defects in bone filled in perfectly well without any aid, and our present data show that even worryingly large defects will reform. There is, however, a strong correlation between the risk of subsequent complications such as fracture or osteoarthritis and the size of the cyst at the time of diagnosis. In our experience, cysts larger than 60 cm^3^ (i.e. cysts of 5 cm or more) have the greatest risk of complications and cysts smaller than this have a low risk.

Apart from bone cement, most substances that are used to fill defects of this sort have no inherent strength and rely upon bone ingrowth for strength (e.g. cancellous allograft, cancellous autograft, most bone substitutes). Thus, these patients will be as much at risk of fracture or collapse of the joint surface as those without any filling, until the bone has consolidated. Again, there is no evidence to show that this happens more quickly if bone graft or bone substitute is used.

None of the currently available materials for filling bone defects of this sort are without problems. We have shown that small defects (under 60 cm^3^, i.e. approximately less than 5 cm) have few problems if they are left empty, and so it is only the larger defects that are likely to need filling. Obtaining more than 60 cm^3^ of autograft is quite a large operative procedure, which is likely to lead to significant morbidity. There may be reluctance to use allograft, however, particularly in children or young people. Bone substitutes have become more and more popular—but with little evidence of their efficacy, particularly in defects of this size (Buma et al. 1996, Finkemeier 2002).

**Figure:**
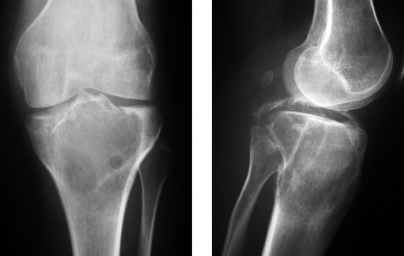
A 25-year-old woman with cylinder form giant cell tumor of 220 cm^3^. There was an intra-articular fracture preoperatively. Full weight bearing was allowed at 6 weeks after curettage.

**Figure:**
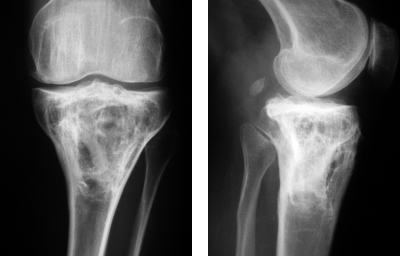
The same patient 9 years after surgery. She developed osteoarthritis during the follow-up.

Bone substitutes might be safer, in the sense that they do not transmit any viral diseases (except substitutes that have human or animal origin). It is often forgotten, however, that the most common life-threatening complication is infection and any foreign material can increase the risk of transmitting bacterial infection (Aspenberg 1998).

The market is full of many different kinds of bone substitutes only waiting for a cyst to be filled. In the literature, most bone substitutes are compared with some other filling material under optimal conditions. Many of these porous substitutes have numerous advantages over autografts and allografts because of their unlimited supply, and easy sterilization and storage (Bucholz 2002), but most are quite expensive—particularly so when used to fill large defects. Most of them seem to incorporate well, and if structural support is not required the function is almost comparable with autograft for bone filling purposes. There has, however, been a lack of studies in which bone substitutes have been compared to the spontaneous healing of bone. The blood circulation is good in cancellous bone, promoting spontaneous regeneration. Following a detailed curettage, the margins of the defect will always contain healthy cancellous bone—which will allow migrating osteoblasts to produce bone matrix in the haematoma that will fill the defect, a scenario very similar to that in fracture healing.

There are experimental data showing that bone defects that are left empty heal just as well as when filled with a bone substitute (Medige et al. 1982, Virolainen et al. 1997, Lemperle et al. 1998). In our study, we did not obtain any histological evidence of new bone formation in the cavities, although the plain radiographs demonstrate that the cavities were still visible even years after surgery (Figures). Even if the bone cavity did not reform completely, the cortical bone was thick enough to carry the load. Inside the cavity, there were smaller cavities divided by several new septa.

All the postoperative pathological fractures happened during the first weeks after the surgery, and not when full weight bearing was allowed. This was also found to be the case in the experimental model of large persistent cysts (Medige et al. 1982). Full weight bearing and mechanical stress finalize the strength and the healing process of the injured bone (Medige et al. 1982, Isaksson et al. 2005).

There is a need for prospective randomized studies to evaluate the effect of different filling materials on the remodeling of bone. Our data provide a basis for further studies involving the treatment of benign bone cysts and suggest that these studies should concentrate on cysts larger than 5 cm.

We conclude from this study that most benign defects of bone will consolidate without supplementation.
